# Remote Fieldwork With African Migrant Women During COVID-19 Pandemic in London: A Reflection

**DOI:** 10.3389/fsoc.2022.788180

**Published:** 2022-04-04

**Authors:** Cathrine Madziva, Martha Judith Chinouya

**Affiliations:** ^1^Department of Health, London Metropolitan University, London, United Kingdom; ^2^Liverpool School of Tropical Medicine, Liverpool, United Kingdom

**Keywords:** remote fieldwork, ethics, COVID-19 pandemic, socially disadvantaged groups, crisis response plan, African migrant women

## Abstract

As coronavirus disease 2019 (COVID-19) pandemic unraveled, state-led preventative restrictions created a “new” normal through remote home-working. A long-planned follow-up qualitative research study on risk perceptions and experiences regarding Clay Ingestion among black African women during pregnancy, in London, was disrupted as England went into lockdown. Against this backdrop, we shifted to remote data collection which raised pertinent concerns around access to technology and participant digital skills. We share our experiences of navigating through remote fieldwork during the pandemic with black African mothers with caring responsibilities as well as the extra burden of homeschooling, the challenges we encountered and how we mitigate these and the lessons learnt. Thus, drawing from our remote qualitative research experiences, we refer to notable examples of challenges, mitigating strategies applied and potential lessons to inform future practice.

## Introduction and Background

As coronavirus disease 2019 (COVID-19) began to unravel in England, we had previously conducted a study with black African women exploring late access to antenatal care services and geophagy (clay ingestion) during pregnancy emerged as an important theme (Chinouya and Madziva, 2017; Madziva and Chinouya, 2020). Findings from this study, and elsewhere (Abrahams et al., 2006; Frazzoli et al., 2016), indicated women ingested clay to help them cope with some of the challenges associated with pregnancy such as nausea. While clay ingestion is perceived “acceptable” and culturally embedded in many African countries such as Nigeria, Ghana, Kenya, Zimbabwe, South Africa, Uganda (Njiru et al., 2011; Henry and Cring, 2013; Frazzoli et al., 2016), its discovery in this diverse population group in some European countries has evoked the interests of biomedical scientists to investigate the make-up of clay contents (Abrahams et al., 2006; Reeuwijk et al., 2013).

In the UK, the detection of high levels of lead and arsenic in clay intended for ingestion led the Food Standards Authority (FSA) to issue repeated warnings between 2011 and 2012 (Food Standards Agency, 2012). This was followed up by Public Health England's directive to general practitioners, directors of public health, and other public health practitioners to dissuade pregnant women from ingesting what the agency described as a potentially “poisonous product” (Public Health England, 2013). This is not without good reason; extensive scientific evidence suggests that persistent exposure to high levels of lead and arsenic found in clay products during pregnancy can lead to low birth weight, impaired intrauterine growth, impaired neurodevelopment, and intestinal blockages (Reeuwijk et al., 2013; Nyanza et al., 2014; Gundacker et al., 2017). Lower levels of exposure to lead are now known to affect children's brain development resulting in reduced IQ and attention span, antisocial behavior as well as reduced educational attainment (WHO, 2021).

Despite these repeated health warnings, there is evidence that clay ingestion remains an important aspect of pregnancy among African communities (Madziva and Chinouya, 2020). Against a backdrop of this disconnect—between official public health messages and ground realities—we designed a qualitative study that aimed to explore clay ingestion experiences and risk perceptions using face-to-face semi-structured interviews and a focus group discussion (FGD). COVID-19 preventive measures brought in by the UK government rendered physical contact and face-to-face data collection impossible and as a consequence, we had to conduct the study remotely.

In the context of public health and research, migrants are often classified as “hard to reach” due to difficulties in accessing or involving them in research (Chinouya and Madziva, 2017). This partly stems from being socially disadvantaged (Lambert and Wiebel, 1990; Sydor, 2013) as well as reluctance to be officially contacted (Shaghaghi et al., 2011). According to Witham et al. (2020), COVID-19 pandemic has made it more challenging to access this population group. While most academic institutions were able to adopt remote working and delivery due to prior investments in IT infrastructure systems (Dhawan, 2020) engaging local communities in remote fieldwork has been fraught with challenges. Mitchell (2021) cites the lack of digital skills or access to technologies, loss of child care and support as well as the social and economic deprivation, economic shocks, and ill health brought on by the pandemic as key areas of concern. During the time, we conducted the study (May–August 2020) we found ourselves navigating through a fluid and challenging landscape with very limited informing academic literature bar a number of emerging blogs, albeit all lacking in practice. Since then, there have been a few academic articles, e.g., Reñosa et al. (2020) informing qualitative research practice in the face of an ongoing pandemic albeit focusing on experiences gleaned from other countries and not on migrant communities.

Against this background, we aim to share our experiences of navigating through remote fieldwork during the pandemic with Black African mothers with caring responsibilities as well as the extra burden of homeschooling. Thus, drawing from our remote qualitative research experiences, we refer to notable examples of challenges encountered, mitigating strategies applied and lessons learnt to inform future practice. Noteworthy is that this reflection does not focus on reporting a qualitative research study or its central underpinnings, but methodological and practical experiences drawn from remote recruitment and sampling as well as data collection. Evidence suggests that socially disadvantaged groups, mostly black and ethnic minority communities bore the economic brunt of the pandemic (The Migration Observatory, 2020) and on this note, we also share how a £15 voucher given to participants for participating in the study unexpectedly presented a distressing snapshot of deprivation.

## Remote Participant Recruitment and Sampling

As noted by Reñosa et al. (2020: 1) “face-to-face interaction is the hallmark of qualitative research data collection as this enables rapport building, open and honest dialogue with research participants as well as showing empathy.” Our prepandemic plan had been to recruit self-identifying black African women who had ingested or were ingesting clay during pregnancy in inner London Boroughs at places they are known to frequent such as churches, community centers, African markets and shops, hairdressing salons, mosques inter alia through convenient, opportunistic and snowballing techniques. Remotely recruiting a hard-to-reach population group posed several challenges for us. COVID-19 prevention guidance of not socializing with people outside one's household led to the loss of liberty to directly recruit participants from places they are known to frequent. Our attempt to use social media platforms, such as Facebook, to recruit was short lived and not very successful. This was potentially due to the sensitivity of the topic as well as the target population being suspicious of real and imagined authorities, especially the UK Border Agency (Chinouya and Madziva, 2017). This left us relying heavily on snowball sampling to recruit participants—a method we had successfully relied on in a previous study.

Snowballing is defined as a sampling method whereby the sample is built up by recruiting potential participants from known informants (Ritchie et al., 2013). To start the snowballing, we engaged two black African community mobilizers and trained them to be “online recruiters” using the study participant information sheet. It was important for the recruiters to be “insiders” in terms of having a loosely shared identity and cultural background with participants for the purposes of building trust and rapport. Participants were selected with a purpose to “represent” the criteria which included the following:

- Self-identifying as black African.
- Over the age of 18 years old.
- Living in a London borough.
- Having experienced clay ingestion during pregnancy in England in the last 10 years.

The snowballing aspect involved community mobilizers asking each interviewed participant to identify and refer others they knew to fit the selection criteria. As Ritchie et al. (2013) note; snowballing sampling works well for recruiting dispersed and small population groups with selection criteria that may not be as widely disclosed due to topic sensitivity. However, recruiting new participants from an existing sampling pool risked the sample's diversity as sampling members would refer a potential participant from the same country of origin. While the standardized term “Black African” does, on face value allude to a homogeneous group, we were acutely aware of the contestable nature of this label and its' potential to mask the heterogeneity of black Africans (Aspinall and Chinouya, 2008). To mitigate this, we introduced quota sampling.

Quota sampling entails recruiting participants till a certain quota is reached (De Vaus, 2013). By introducing this technique, there was no intention to make the sample statistically representative which is often the case with quantitative research, but to improve the sample diversity as much as possible. With a planned sample of thirty, once we had recruited three or four women from the same country, we requested sampling pool members to refer participants from a different country of origin. In addition, we used “links.” These were people known to us researchers and community mobilizers but crucially did not fit the selection criteria. Their role was to refer women known to meet the criteria. While this was cumbersome, it improved the sample diversity as well as widened the distance; to some extent, between the sample pool, i.e., from close family and friends' social circles (Ritchie et al., 2013).

The first stage of recruiting participants known to recruiters and researchers was straightforward because contact details were readily available. Thus, researchers and community mobilizers were able to call potential participants and explain the study as well as send vital study information to those who showed interest in taking part. However, recruiting new participants from our existing sampling pool and “links” had additional challenges because we had to rely on them to initially explain the study. The lack of direct access to potential participants cost us opportunities to accurately explain the study, its importance as well as address arising questions. To ensure potential participants had sufficient information to agree to being contacted by the research team; community mobilizers and researchers encouraged sampling pool members and “links” to share the study information sheet, which largely depended on their goodwill. The topic area however resonated with potential participants, who had not “imagined” it could be a topic of academic research hence even those who had barely understood the study and or received sketchy information were interested enough to agree to being contacted by the research team. In this study, community mobilizers had well-established connections and networks across different London boroughs which proved pivotal to a successful recruitment drive. Our experience suggests that community mobilizers can play a key role in recruiting hard-to-reach population groups, particularly in the presence of a shared identity with potential participants.

## Ethics, Building Trust and Rapport, and Data Collection

This study was granted ethical approval by London Metropolitan University Ethics Review Committee. As the pandemic unraveled, we had to consider the health and well-being of research participants first. Hence, from the onset, we had to ensure participating in the study would not place undue stress on participants. We achieved this by stressing the voluntary nature of participation, as well as allowing participants to decide when the interviews took place. Obtaining written and signed consent remotely however proved problematic for us due to a number of reasons. Unable to issue hard copies in-person or *via* postal services for a physical signature (given participants' suspicion of authorities, we aimed to minimize the collection of personal information, home addresses in particular) we had to rely on discussing the consent form remotely as well as emailing it back and forth. While most participants were able to download the consent form and return it signed, quite a few had challenges. This stemmed from the lack of know-how (sufficient digital skills to competently use the internet to access the document and respond) and not having access to an appropriate device or limited access to the internet. To mitigate the lack of know-how; we sent the consent form as part of the email text and asked participants to consent by responding to the email with a declaration they had read the consent form and agreed to take part in this study. However, in some cases, participants were sharing devices such as smartphones with their children who were homeschooling. This delayed the start of interviews as we had rescheduled at times more than twice. Similarly, a study of parents and children by Ofcom (2021) highlighted that some financially vulnerable households solely relied on mobile internet access with children having to share devices with other family members to engage in home-schooling. While evidence shows a narrowing of the digital divide during the pandemic due to more people turning to online shopping, banking, etc. (Ofcom, 2021). Our experiences in this study highlight the existence of digital poverty within a population group already known to be socially disadvantaged (Jivraj and Khan, 2013; The Migration Observatory, 2020). With the benefit of hindsight, it would have been worth considering oral consent which can be recorded for those with limited digital skills or access to technology. This however requires consideration at planning stages for inclusion in the ethics review process for approval.

We conducted 30 individual interviews with Black African mothers (age ranged from 30 to 45 years old) from Zimbabwe, Uganda, Cameroon, South Africa, Ghana, Democratic Republic of the Congo, Nigeria, Congo—Brazzaville, and Guinea-Bissau and one FGD with 7 participants. The FGD participants were drawn from those who had been individually interviewed. The rationale for a FGD after interviews was to clarify issues emerging from interviews as well as exploring shared “frameworks of understanding” (Carter and Henderson, 2007: 222) on the clay ingestion practice. Each interview lasted approximately 40–60 min and was scheduled around participants' availability; evenings and weekends were included. However, parental responsibilities with home schooling at the fore had us rescheduling several times. Furthermore, it was not uncommon for phone calls to go unanswered. We attempted to mitigate this by sending text message reminders and asking participants to get in touch when able to do so. From an ethical perspective, we were mindful of the time constraints participants faced, hence restrained from adding undue pressure by making multiple calls. Interviews were also disrupted by young children crying and needing parental attention. Prioritizing the need for parents to attend to their children meant that we had to suspend interviews until they were next available. Here, we learnt that exercising patience and flexibility can go a long way in enabling successful data collection while putting the needs of participants first. Researchers would do well to consider allocating generous research time frames at planning stages as this affords some room to maneuver thereby allowing participants to balance out study participation with other time competing claims.

As qualitative researchers, being able to build rapport is central to participants opening up to sharing their experiences and perspectives. We were concerned that remotely interviewing participants who had never met us in person would negatively impact this; however, we found that self-identifying as black Africans with participants helped to build rapport and trust. Throughout the interviews, participants queried our specific countries of origin after discerning our “Southern African accents.” This shared “African identity” meant we had a loosely shared “cultural framework” as well as a shared gender identity that made participants feel at ease and open up. In the absence of this, cultural mediators as successfully utilized by Walker et al. (2021) in their qualitative study with a refugee and asylum seeker participant group may be worth considering.

While participants were generally sensitive about “outsiders” from the “African community” (primarily white people) knowing, they ingested clay, there were not minded to discuss this within earshot of their family members. Even as we highlighted the need for privacy and confidentiality before each interview, participants often pointed out that family members were already privy to their clay ingestion with many acting as enablers by sourcing the clay on their behalf. Facilitating the FGD which lasted 1 h and 45 min was, however, most challenging because of participants' availability at different times. We eventually settled for a Saturday afternoon using WhatsApp—a popular application familiar to all participants and which they already had installed on their mobile telephones. In addition, its privacy features were also satisfactory. While we had initially planned to use the audio only, participants expressed the desire to see the facilitator (one of the researchers) as well as the other participants taking part in the study. This was partly influenced by the fact that it was a weekend, i.e., they had a break from homeschooling. Having conducted mobile telephone individual interviews with some of the participants busy doing house chores such as ironing, dishwashing, cooking, we had concluded a video FGD as too disruptive. We went along with the video preference and participants were able to build rapport with each other very quickly. They were also happy to finally “meet” other participants as well as the facilitating researcher which helped with building synergies, a key ingredient to exploring shared experiences (Carter and Henderson, 2007). On this note, it goes without saying that video calling enables some degree of in-person advantages to be replicated. Hence, we were able to observe crucial non-verbal communication, i.e., body language and other cues which helped us with further probing. Hence, our takeaway here is that it is best to plan for a video discussion from the onset; even when only recording the audio aspect (as in our case). With flexibility in mind, it is worth weighing when participants have less shared time competing claims as this is likely to have a bearing on this preference.

## “Where is the Voucher? I Need it to Buy Food for My Children…”

While there are different forms of payments that can be made to research participants with varying ethical implications, in this study a £15 E-voucher payment for Supermarket X was made in acknowledgment of each participant's time, experiences, and knowledge. This was initially meant to be in cash, but due to the pandemic, we had to rethink this. As the University of Oxford Central Research Ethics Committee notes, for the socially disadvantaged groups, this can afford them the dignity of rewards for their contributions (University of Oxford, 2020). While it is beyond the remit of this reflection to engage with the wider ethical debates around payments, in this study, we informed participants of the voucher payment after they had agreed to take part in the study. This was because the payment was not an incentive meant to influence them to participate, but rather an acknowledgment of their contribution as well as time taken out to participate. This is mitigated against participants agreeing to take part when they would otherwise not have which is often an ethical concern (Ibid.).

However, when participants were emailed the e-voucher as a live link along with instructions on how to open and use it, two issues emerged: a few of the participants—from among those who had limited digital skills—had difficulties in opening this. We rectified this by explaining the process step by step over the phone until they successfully accessed the e-voucher. Notwithstanding this, what caught us unprepared, was how the voucher laid bare the economic challenges experienced by some of the participants. While we had informed, them they would receive the voucher within 2 weeks of interviews, some participants would call asking for the voucher before the agreed timeframe. We received queries^1^ such as: “Where is the voucher? I need to it to buy food for my children”; “I am following up on the voucher, I need it to buy food.” One particular participant lamented: “I am in supermarket X with groceries in my basket. I cannot find the email with the voucher and I have no money to pay.” While the voucher had already been sent to her, but had landed in her email junk folder which she had not checked, the likelihood of walking out of the supermarket without the groceries had caused her a great deal of distress.

In our research ethics application, we had given due diligence to the potential risks and having a response plan which would signpost those unwell or concerned or anxious about their health with regards to the pandemic and clay ingestion to health services as well as a well-known community organization. We had not however anticipated a situation where the voucher would make a difference between having a meal or not. This was distressing and left us questioning our preparedness to respond to such situations. With a combined experience of over 25 years of conducting qualitative research and research training at a doctorial level between us, we had not encountered this level of need in similar previous sample pools. This was a novel experience that we attributed to the pandemic's impact on those already socially disadvantaged. Evidence shows that as COVID-19 hit, low-income earners such as those in our sampling pool, bore the brunt with families turning to food banks to make ends meet (The Trusswell Trust, 2021). More than half of our sample members were either care workers or services industry workers on zero contracts who had instantly lost their income. For some, the inability to work (partners included) due to either shielding or isolating or job loss had left them in severe economic hardship. Most telling for this study sample constituting of black African women with young children is evidence which suggests that families with children were the hardest hit making up about 40% of those who needed food support; with those identifying as Black or Black British significantly over presented in food bank usage (The Trusswell Trust, 2021). While the government stepped in to provide support through the Furlong scheme and £20 increase to universal credits, nearly 1.4 million people who live in the UK with no recourse to public funds (NRPF) fell through the net (The Migration Observatory, 2020). As ibid., further notes; NRPF restrictions fall disproportionately on ethnic minorities. Findings from a previous study (Chinouya and Madziva, 2017) among black African women in London showed the existence of mothers lacking legal status to remain in the country. This left them in a precarious position as they stayed away from antenatal services till birthing time for fear of UK border Agency officials. The lack of legal status automatically translates to NRPF restrictions. In the context of this study, the issues raised around the voucher suggest that the pandemic left some families with children in this category more vulnerable than ever before.

While ethical issues are encountered at all stages of the research process, quite often researchers are able to address most of these preemptively during the ethics approval stage through risk mitigating strategies. However, our experience with the voucher suggests that conducting research during a pandemic can generate a host of ethical issues, albeit not necessarily novel but not well evidenced or acknowledged. The relative stability of developed countries as research settings in comparison to disaster or humanitarian crisis settings in developing countries where potential risks may be more visible can mask vulnerabilities within the socially disadvantaged groups who may lack entitlement to government welfare support. It is, therefore, imperative that in the context of a pandemic, settings like these are subjected to extra scrutiny in terms of potential risks faced by participant groups already known to experience higher levels of inequalities. This can go a long way in enabling researchers to develop crisis response plans that are responsive to critical situations on the ground as and when these arise. Notwithstanding, we remain concerned that conducting research with socially disadvantaged groups during a pandemic is more likely to raise ethical issues than those addressed at the ethics approval stage because of the fluidity of ground realities. In our case, the voucher issues we have raised left us distressed.

## Conclusion

Drawing from our remote qualitative research experiences, this reflection has shared notable examples of challenges, mitigating strategies applied and potential lessons to inform future practice. We conclude that while it was possible to conduct remote fieldwork with a socially disadvantaged group during a pandemic, patience, flexibility, and general awareness of competing time claims on the participant group are key requirements. This has implications on research time frame allocations from the onset. While there are challenges with recruiting, building rapport and trust with participants never met in person, engaging with community mobilizers as well as having a shared identity with participants can go a long way in mitigating these challenges. Remote fieldwork can also raise challenges with the management of consent forms when dealing with participants with limited digital skills or access to technology. This requires thinking outside the box, for instance, the use of oral consent which can be recorded. Lastly, conducting research during a pandemic can generate a host of poorly evidenced ethical issues. While we recommend subjecting research settings to extra scrutiny to inform crisis response plans, we also remain concerned that the fluidity of ground realities potentially raises more ethical issues than those anticipated for at the ethics approval stage. Against this backdrop, we argue that there is a need to build an evidence base regarding those poorly evidenced ethical challenges researchers face in the course of knowledge generation.

Furthermore, research that focuses on those challenges and how they can have mitigated in different research settings is needed.

## Author Contributions

All authors listed have made a substantial, direct, and intellectual contribution to the work and approved it for publication.

## Funding

The study upon which this reflection is based was funded by the Rescaling Fund, London Metropolitan University.

## Conflict of Interest

The authors declare that the research was conducted in the absence of any commercial or financial relationships that could be construed as a potential conflict of interest.

## Publisher's Note

All claims expressed in this article are solely those of the authors and do not necessarily represent those of their affiliated organizations, or those of the publisher, the editors and the reviewers. Any product that may be evaluated in this article, or claim that may be made by its manufacturer, is not guaranteed or endorsed by the publisher.

## Footnotes

^1^Remote field note.
